# AQQD: Annotated Quranic Qira’at Dataset

**DOI:** 10.1016/j.dib.2026.112972

**Published:** 2026-06-11

**Authors:** Linda Smail, Mohammed Lataifeh, Md Sohazur Islam Sozib, Arthur Diniz De Souza

**Affiliations:** a Mathematics Department, Zayed University, Dubai, 19282, United Arab Emirates; b Department of Computer Science, University of Sharjah, Sharjah 27272, United Arab Emirates

**Keywords:** Quran, Recitation, Audio, Classification, Speech processing, Phonetic variation, Machine learning

## Abstract

AQQD (Annotated Quranic Qira'at Dataset) is an open audio dataset of Quranic recitations annotated across canonical Qira'at styles. The dataset is designed to support research in machine learning, speech and audio processing, computational linguistics, and Quranic studies. The current release contains 24,183 WAV audio files from 309 reciters and covers 70 selected Quranic Surahs segmented into representative verses and phonetic variation points. Of these, 23,111 recordings were collected from publicly available sources, including official reciter websites, the Midad repository, MP3Quran, and verified YouTube channels, while an additional controlled subset of 1,072 recordings was obtained from a single reciter recorded as a Mus'haf provided by Taibah University (Madinah, Saudi Arabia). This controlled subset enables comparative analysis across Qira'at styles while minimizing speaker variability. Recordings are standardized to 44.1 kHz, 16-bit, mono WAV format and range from 6 to 17 seconds in duration. Structured metadata is embedded directly in filenames, enabling efficient filtering, indexing, and preprocessing. The dataset is publicly available under a CC0 1.0 Public Domain license and supports a range of reuse scenarios, including Qira'at classification, phonetic variation analysis, recitation verification, speaker-independent audio modeling, and the development of educational technologies for Quranic studies.

Specifications Table

**Table:** 

Subject area	Computer Science (Machine Learning); Islamic Studies (Quranic and Qira’at Studies)
Specific subject area	Computational Quranic studies; Speech/audio processing; Qira’at classification
Type of data	Audio dataset (WAV recordings); Metadata (structured filenames and annotation tables).
How data were acquired	Audio data were collected from publicly available sources including official reciter websites, the Midad repository, MP3Quran, and verified YouTube channels. An additional controlled subset of 1,072 recordings was obtained from a single reciter provided by Taibah University (Madinah, Saudi Arabia).
Data format	Audio files are provided in uncompressed WAV format (44.1 kHz, 16-bit, mono), each named according to a structured convention indicating the reciter, recitation style, surah, aya, and clip number. The clips range in duration of approximately 6-17 seconds.
Data source location	Primary data sourcing, updating, and comparative validation were carried out within Zayed University, United Arab Emirates, serving as the central hub for collecting, organizing, and verifying recordings from all trusted sources used in the dataset.
Data accessibility	Available at Harvard Dataverse: Smail, L. (2026), “AQQD – Annotated Quranic Qira’at Dataset”, https://doi.org/10.7910/DVN/A8GM5Y, under the CC0 1.0 Public Domain license.

## 1 Value of the Data

• AQQD is, to the best of our knowledge, the first openly accessible dataset that systematically captures multiple canonical Quranic Qira'at styles within a unified and standardized audio collection, addressing an important gap in computational Quranic studies and speech/audio processing research.
• The dataset combines 23,111 recordings collected from diverse public Quranic recitation sources with an additional controlled subset of 1,072 recordings from a single reciter provided by Taibah University, enabling both speaker-diverse and speaker-controlled comparative analysis across Qira'at styles.
• Recordings are standardized to 44.1 kHz, 16-bit, mono format and include structured metadata embedded directly in filenames, enabling efficient filtering, indexing, preprocessing, and reproducible experimentation.
• The dataset supports multiple research and educational applications, including Qira'at classification, phonetic variation analysis, recitation verification systems, educational technologies, and computational studies of Quranic oral transmission.
• The inclusion of multiple reciters per Qira'at style improves model robustness and reduces speaker-specific bias, supporting the development of more generalizable AI and audio analysis frameworks.
• AQQD supports several benchmark tasks and experimental designs, including automatic Qira’at classification, speaker-independent Qira’at recognition, phonetic variation detection at Qira’at-specific variation points, and recitation verification systems. The dataset can be used to evaluate model generalization across reciters, investigate acoustic differences among canonical Qira’at styles, and develop educational and research tools for analyzing Quranic recitation.

## 2 Background

While the Holy Quran is commonly encountered today in written form, the science of qira'at is fundamentally grounded in oral transmission. The Quran was revealed as recited speech, and its preservation has historically depended on direct auditory transmission (mushafahah) through authenticated chains of narration (isnad) [1]. The written muṣḥaf serves primarily as a referential aid; authoritative knowledge of recitation resides in sound rather than in script. As Nelson [2] emphasized, the Quran is not fully realized unless it is heard, since intonation, rhythm, articulation, and timing are integral to its meaning and religious function.

**Fig. 1:**
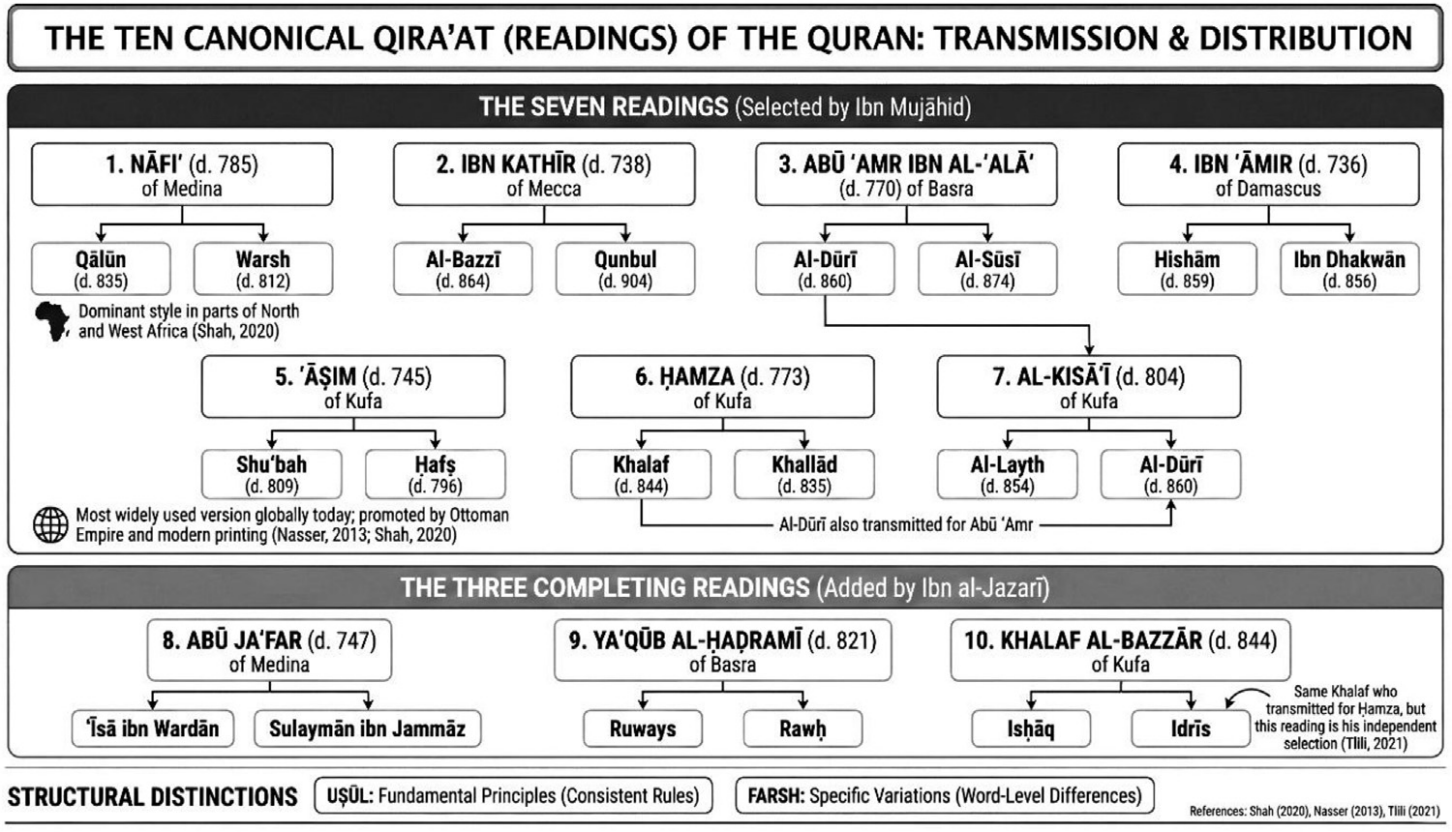
The ten canonical Qirāʾāt of the Qurʾān: transmission and classification according to Ibn al-Jazari.

This oral foundation gave rise to multiple valid modes of recitation, known as qira'at, representing canonically accepted schools of pronunciation and vocalization. Ibn Mujahid later canonized seven readings, and Ibn al-Jazari expanded them to the ten canonical qira'at recognized today [3,4], as shown in Fig. 1.

Differences among qira'at include phonological variations such as imala, idgham, madd, and hamzah realization, as well as specific lexical variants preserved through oral transmission [5]. These distinctions are fundamentally acoustic rather than textual, making qira'at particularly relevant for speech processing and computational audio analysis.

To the best of our knowledge, AQQD is the first comprehensive annotated dataset featuring multiple canonical Quranic Qira'at. While previous Quranic audio datasets such as Ar-DAD focused primarily on reciter identification and general Quranic speech processing [6], AQQD specifically captures authenticated variation across canonical Qira'at styles, providing a standardized resource for machine learning, phonetic analysis, and computational Quranic studies.

## 3 Data Description

### 3.1 Data acquisition and audio standardization

Audio data were collected from publicly available, trusted online sources, including official pages of certified Quran reciters, the Midad Quran Audio repository [7], the Holy Quran Recitation Archive-MP3Quran website [8], and verified reciters' YouTube channels. Recordings were curated, segmented, and standardized to ensure consistent quality and coverage across Qira'at styles. All selections were manually reviewed to confirm authenticity and correct labeling of each recitation style. Primary data sourcing, updating, and comparative validation were carried out at Zayed University, United Arab Emirates, which served as the central hub for collecting, organizing, and verifying recordings from all sources.

### 3.2 Dataset repository and organization

The AQQD dataset has been deposited in the Harvard Dataverse repository (Dataverse ``AQQD'') under the CC0 1.0 Public Domain license and is publicly accessible via its permanent DOI https://doi.org/10.7910/DVN/A8GM5Y [10]. The repository contains the complete collection of 24,183 audio recordings distributed across compressed archive files (AQQD V2.0.zip.001–011), along with accompanying metadata (AQQDV2 details.xlsx) and documentation (README_FIRST.txt). Users are advised to consult these resources for dataset structure, extraction procedures, file organization, and usage instructions.

The release includes 23,111 recordings collected from public repositories and an additional controlled subset of 1,072 recordings obtained from a single-reciter recorder as Mus'haf provided by Taibah University (Madinah, Saudi Arabia).

### 3.3 Dataset structure and coverage

The dataset is conceptually structured into three content tiers based on the selection strategy for Quranic passages, but these tier labels are not reflected in the directory structure or filenames. In total, AQQD covers 70 surahs (chapters) of the Quran, each represented by one or more specific verse segments, which are then recited in multiple canonical Qira'at styles. This three-tier structure ensures inclusion of both frequently recited passages and those that exemplify characteristic Qira'at differences:

—Tier 1 – Core Short Surahs: Complete short chapters of the Quran that are foundational in daily recitation (e.g., Al-Fatiha 1:1–7, Al-Ikhlas 112:1–4, An-Nas 114:1–6). These 19 surahs are fully included in all available Qira'at styles and serve as a consistent baseline for comparative analysis across recitation traditions.
—Tier 2 – Representative Long/Medium Surahs: Selected segments from 30 surahs of medium to long length, each represented by typically an opening, middle, and closing passage (e.g., Al-Kahf 18:1, 18:50, 18:100, last; Ya-Sin 36:1, 36:36–40, last). This structure captures variety in linguistic context and melodic cadence without requiring the recording of entire lengthy chapters.
—Tier 3 – Special Qira'at Points: Verses or short passages (21 in total) known for Qira'at-specific variation, such as As-Sajdah 32:15, Al-Ahzab 33:56, and Al-Qalam 68:1–4. These selections highlight subtle phonetic or textual differences—e.g., imala (vowel tilting), handling of hamzah, or madd (vowel lengthening)—across canonical reading styles, providing high-value material for detailed comparative analysis.

Across all tiers, the dataset spans 70 unique surahs out of the 114 in the Quran, encompassing both Makki and Madani chapters and a broad range of lengths and themes. Each selected passage has between six and eight Qira'at renditions, depending on the availability of qualified reciters. Canonical readings represented include Ḥafṣ ʿan 'Āṣim, Warsh ʿan Nāfi', Qalūn ʿan Nāfi', Ad-Dūri ʿan Abū 'Amr, among others. The current release expands coverage of the ten canonical Qira'at by including Yaʿqūb al-Ḥaḍramī and Khalaf al- 'Āshir (Khalaf al-Bazzār). Table 1 provides an overview of the tiered content selection strategy in AQQD.

**Table 1:** Overview of the tiered content-selection strategy in AQQD.

Tier	Description	Number of Surahs/Passages	Coverage Purpose
**Tier 1**	Complete short surahs commonly used in daily recitation (e.g., Al-Fatiḥa, Al-Ikhlaṣ, An-Nas)	19 complete surahs	Baseline comparison across Qira’at
**Tier 2**	Selected segments from medium and long surahs (opening, middle, closing passages)	30 surahs (partial coverage)	Linguistic and melodic diversity
**Tier 3**	Verses with known Qira’at-specific variation (e.g., *imala, hamzah, madd*)	21 targeted passages	Fine-grained Qira’at analysis

Table 2 presents the distribution of recordings across the ten canonical Qira'at styles represented in AQQD. As expected from differences in source availability and reciter accessibility, coverage remains uneven across some styles. Researchers developing machine-learning models should account for this class imbalance during training and evaluation by using appropriate class weighting, resampling strategies, or stratified validation procedures.

**Table 2:** Distribution of recordings across the ten canonical Qira’at styles represented in AQQD.

Code	Qira’at Style	Files	Percentage (%)
S1	Nāfi' al-Madanī	7488	30.96
S2	Abū 'Amr al-Baṣrī	3463	14.32
S3	Abū Ja'far al-Madanī	79	0.32
S4	Ibn Kathīr al-Makkī	3025	12.51
S5	'Āṣim al-Kūfī	7571	31.33
S6	Al-Kisā'ī	173	0.72
S7	Ibn 'Āmir al-Shāmī	1466	6.05
S8	Ḥamzah al-Kūfī	799	3.30
S9	Yaʿqūb al-Ḥaḍramī	57	0.24
S10	Khalaf al-'Āshir	60	0.25
Total		24,183	100

### 3.4 File naming convention and metadata structure

Each recorded recitation in AQQD is provided as an individual audio file in uncompressed WAV format (44.1 kHz sampling rate, 16-bit depth, mono). Mono audio was used to ensure consistent acoustic features across sources, as spatial/stereo information is not relevant to Qira’at classification, phonetic analysis, or the traditional oral transmission modeling. The clips range from 6 to 17 seconds in length, depending on the passage. Filenames encode key metadata following a structured convention that identifies the reciter, recitation style, surah, aya, and clip number (e.g., R012_S3_Surah_005_Aya16_C2.wav). Each file name follows a fixed schema:

R[ReciterID]_S[QiraatID]_Surah_[SurahNumber]_Aya [AyaNumber]_C[ClipNumber].wav

**Fig. 2:**
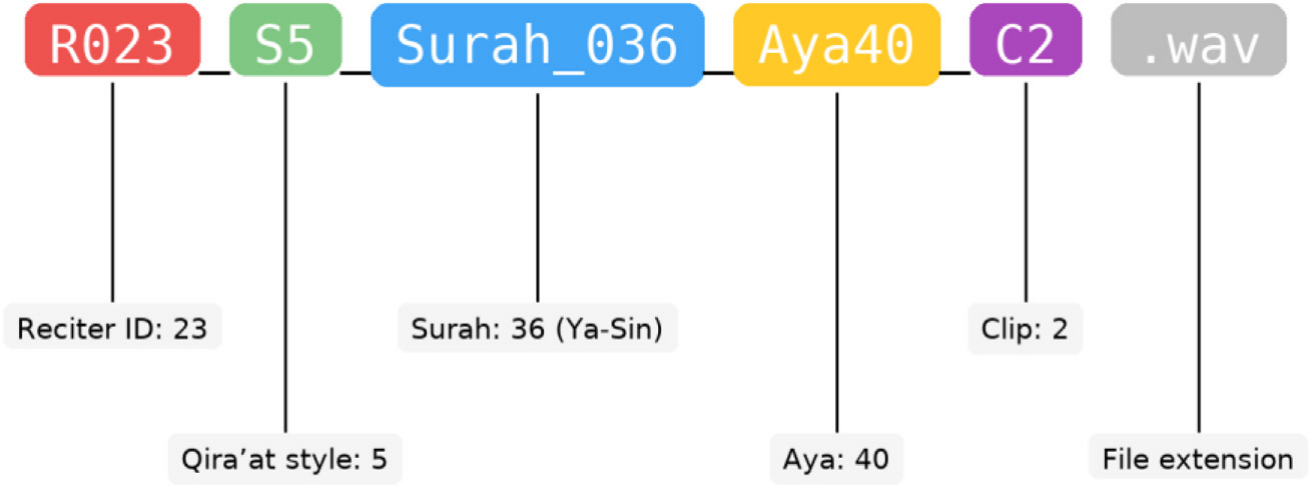
Filename structure in AQQD, illustrating the encoded metadata fields within each audio file name. The example R023_S5_Surah_036_Aya40_C2.wav represents Reciter 23, Qira’at style 5, Surah 36 (*Ya-Sin*), Aya 40, Clip 2.

This structured naming convention allows users to extract metadata directly from filenames using automated scripts, without requiring auxiliary metadata files (Fig. 2). For example, the filename R023_S5_Surah_036_Aya40_C2.wav represents Reciter 23**,** Qira’at style 5**,** Surah 36 (*Ya-Sin*), Aya 40, Clip 2. This organization enables flexible data retrieval, such as filtering all samples for a specific reciter, Qira’at style, or surah. It also ensures compatibility with standard data loaders and preprocessing tools in machine learning pipelines.

No separate annotation, transcript, or pre-computed feature files are included in this version of the dataset; these components are not included in the current release. All clips were curated from trusted online repositories, standardized in format, and quality-checked to ensure clarity and consistency across sources. The current data organization emphasizes clarity and interoperability: researchers can parse the structured filenames to group or filter samples by surah, recitation style, or reciter, as needed.

### 3.5 Technical validation

To ensure dataset integrity, reproducibility, and suitability for computational analysis, all recordings in AQQD underwent a multi-stage validation and quality-control process.

#### 3.5.1 Source verification

All recordings were collected from trusted and publicly accessible repositories, including official Quran reciter platforms, the Midad Quran Audio repository, MP3Quran, verified YouTube channels, and the controlled Taibah University Mus’haf subset. Reciter identities and associated Qira’at styles were manually cross-checked against established recitation sources to ensure authenticity and labeling accuracy.

#### 3.5.2 Quality control

Audio files were standardized to a 44.1 kHz sampling rate, 16-bit depth, and mono WAV format to ensure consistent downstream processing and acoustic comparability. Recordings exhibiting severe background noise, clipping, echo, distortion, or incomplete recitation segments were excluded when quality correction was not feasible. During the quality-control stage, approximately 192 candidate recordings were removed for these reasons. The remaining recordings were segmented and normalized to durations from approximately 6 to 17 seconds to maintain consistency across samples while preserving the natural characteristics of the recitations.

#### 3.5.3 Structural consistency check

Automated validation scripts verified filename compliance with the predefined metadata schema and ensured the uniqueness of each (Reciter, Qira’at, Surah, Aya, Clip) combination. Duplicate, corrupted, or structurally inconsistent files were removed prior to release.

#### 3.5.4 Manual expert review

All recordings in the final AQQD dataset (100%) were manually verified by the research team. The review process confirmed reciter identity, Qira’at labeling, pronunciation clarity, segmentation accuracy, recitation authenticity, and consistency with the expected characteristics of each Qira’at style. This comprehensive review complemented automated validation procedures and helped identify potential labeling inconsistencies, segmentation errors, and quality issues before the dataset was released.

Collectively, these procedures improve the reliability, authenticity, and interoperability of AQQD while reducing the likelihood of mislabeled or low-quality samples. As a result, the dataset can be readily used in machine learning, speech-processing, and computational Quranic studies applications without requiring extensive additional preprocessing.

## 4 Experimental Design, Materials, and Methods

### 4.1 Data collection methodology

Source selection prioritized publicly accessible repositories with verifiable reciter credentials and clear Qira'at attribution. Inclusion criteria required that each recording be associated with a named, certified reciter whose Qira'at style could be independently confirmed against established scholarly sources. Recordings were excluded if the reciter's Qira'at attribution was ambiguous, if audio quality was insufficient for reliable acoustic analysis, or if the passage selection did not align with the tier structure described in Section 3.3. The controlled subset from Taibah University (Madinah, Saudi Arabia) was provided as a complete Mus'haf recording by a single reciter proficient in the ten canonical Qira'at styles, enabling direct phonetic comparison across styles under consistent speaker and acoustic conditions. All source materials were reviewed for copyright status and confirmed to be distributed as Islamic endowment materials (Waqf) for educational and research use.

The dataset was constructed using a purposeful sampling strategy designed to maximize coverage of canonical Qira’at variation while maintaining broad representation across the Quran. Rather than randomly selecting passages, recordings were sampled according to the three-tier framework described in Section 3.3, consisting of (i) complete short surahs commonly used in daily recitation, (ii) representative opening, middle, and closing segments from medium and long surahs, and (iii) passages containing known Qira’at-specific phonetic or lexical variations. This sampling strategy ensured coverage of both frequently recited chapters and passages that exhibit distinctive characteristics across canonical Qira’at styles, supporting comparative analysis, speech-processing research, and machine-learning applications.

### 4.2 Audio standardization and processing

Following initial quality screening, during which approximately 192 candidate recordings were excluded because of excessive background noise, clipping, distortion, or incomplete recitation segments, the remaining audio clips were uniformly down- or up-sampled as needed to 44.1 kHz and 16-bit depth and converted to single-channel (mono) format to eliminate channel-dependent variability arising from heterogeneous recording conditions. Each recording was then trimmed or padded to ensure a duration of 6–17 seconds, thereby maintaining consistency across samples.

All audio segmentation and editing were performed using Audacity software [9] for precise control over timing and quality. No additional post-processing, such as noise reduction or normalization beyond the initial standardization, was applied in order to preserve the natural acoustic characteristics of each recitation. All recordings were manually segmented at the verse or phrase level, depending on the location of the Qira’at variation, and subsequently underwent manual verification for segmentation accuracy and labeling consistency prior to inclusion in the final dataset.

No separate transcription or precomputed acoustic feature extraction is included in this first version of the dataset.

Although explicit transcripts and phonetic alignments are not included in the current release, each audio file contains structured metadata embedded within the filename, including the reciter identified, Qira’at style, Surah number, and Aya number (e.g., R023_S5_Surah_036_Aya40_C2.wav). The Surah and Aya identifiers enable straightforward alignment with corresponding Quranic text passages available in standard digital Quranic corpora. Researchers may therefore reconstruct text–audio associations by matching the Surah and Aya fields encoded in the naming schema with the corresponding textual records.

## Limitations

The current release does not include textual transcripts, phonetic alignments, or precomputed acoustic features. Researchers requiring text-audio alignment or detailed phonetic annotation must therefore rely on external Quranic text resources or manually generate additional annotations.

Although recordings were standardized and quality-checked, the dataset was collected from heterogeneous public sources, and some variation in recording environment, microphone quality, and background acoustics may still remain. In addition, coverage across canonical Qira’at styles is not yet fully balanced. Future releases will continue expanding both style coverage and annotation depth.

The current release expands coverage to include all ten canonical Qira’at by including the Taibah University Mushaf recordings. However, coverage remains uneven across some styles due to differences in reciter availability and source accessibility. Despite these limitations, the dataset provides substantial speaker diversity, with recordings from 309 reciters across 70 Quranic surahs, supporting robust comparative and machine learning analyses.

## Ethics Statement

This research was conducted in compliance with institutional and national ethical guidelines. All recordings in AQQD were obtained from publicly available, verified sources featuring certified Quranic reciters. These recordings are distributed as Islamic endowment materials (Waqf) intended for educational and research use. No personal or sensitive data was collected.

## CRediT Author Statement

**Linda Smail:** Conceptualization; Methodology; Data curation; Resources; Software; Visualization; Writing – original draft; Supervision. **Mohammed Lataifeh:** Validation; Methodology; Writing – review & editing. **Md Sohazur Islam Sozib**: Data curation; Software; Validation. **Arthur Diniz De Souza:** Data curation; Software; Validation.

## Data Availability

AQQD (Annotated Quranic Qira'at Dataset) is publicly available through Harvard Dataverse at https://doi.org/10.7910/DVN/A8GM5Y under the CC0 1.0 Public Domain license. The repository contains the complete collection of curated WAV audio recordings, structured metadata files, preprocessing scripts, and accompanying documentation required for dataset exploration and reuse. The current release includes 24,183 audio recordings from 309 reciters, including a controlled subset recorded from a single reciter proficient in the ten canonical Qira'at styles. Detailed usage and extraction instructions are provided in the accompanying README documentation.
